# Co-Adsorption of Alcohols and Water in JUK-8 Studied Using Quasi-Equilibrated Thermodesorption

**DOI:** 10.3390/molecules29102309

**Published:** 2024-05-14

**Authors:** Wacław Makowski, Patrycja Gryta, Gabriela Jajko, Pattaraphon Rodlamul, Damian Jędrzejowski, Kornel Roztocki, Dariusz Matoga

**Affiliations:** 1 Faculty of Chemistry, Jagiellonian University in Kraków, Gronostajowa 2, 30-387 Kraków, Polandgabriela.jajko@doctoral.uj.edu.pl (G.J.); pattaraphon.rodlamul@epfl.ch (P.R.); damian.jedrzejowski@doctoral.uj.edu.pl (D.J.);; 2 Doctoral School of Exact and Natural Sciences, Jagiellonian University in Kraków, Łojasiewicza 11, 30-348 Kraków, Poland; 3 Faculty of Chemistry, Adam Mickiewicz University, Uniwersytetu Poznańskiego 8, 61-614 Poznań, Poland

**Keywords:** adsorption, JUK-8, alcohols, water, quasi-equilibrated thermodesorption

## Abstract

JUK-8 ([Zn(oba)(pip)]*_n_*, oba^2–^ = 4,4′-oxybis(benzenedicarboxylate), pip = 4-pyridyl-functionalized benzene-1,3-dicarbohydrazide) is a hydrolytically stable flexible metal-organic framework. Owing to its unusual adsorptive properties, JUK-8 can be considered as a promising sensing material for construction of detectors of volatile organic compounds (VOCs) in air. Quasi-equilibrated temperature-programmed desorption and adsorption (QE-TPDA) is a versatile method dedicated to characterization of porous materials. In this work, QE-TPDA was employed to study co-adsorption of water and selected alcohols in JUK-8. For the first time an infrared detector sensitive to organic compounds was used in the QE-TPDA measurements, allowing the study of the influence of water vapor on sorption of VOCs. The QE-TPDA profiles of the studied alcohols, exhibiting two desorption maxima and two adsorption minima, are consistent with the standard sorption isotherms, revealing a two-step adsorption–desorption mechanism. The profiles recorded in the presence of water are noticeably changed in different ways for different alcohols. While at low relative humidity (RH) (ca. 20%) the low temperature adsorption states of ethanol and 1-propanol were only slightly destabilized, for 2-propanol almost complete suppression of adsorption was observed. The results found for moderate RH levels (ca. 50%) indicated that the opening of the JUK-8 structure, responsible for its breathing behavior, was followed by the filling of the just generated pores with a water–alcohol mixture.

## 1. Introduction

Flexible metal-organic frameworks constitute an important subclass of porous materials [1,2,3,4,5,6]. Due to phase transitions that involve considerable changes in their crystal structure [7], triggered by various external stimuli, they possess unique adsorption properties, manifested by stepwise changes in isotherms or isobars, often referred to as ‘breathing’ [8,9,10,11]. These effects, resulting from the interactions between the framework and guest molecules, are the premises of numerous potential applications of flexible MOFs in sensing, separation, catalysis, and medicine [6,12,13].

JUK-8 ([Zn(oba)(pip)]*_n_*, oba^2–^ = 4,4′-oxybis(benzenedicarboxylate), pip = 4-pyridyl functionalized benzene-1,3-dicarbohydrazide) is a flexible hydrolytically stable MOF, showing unusual sorption properties [14,15]. The structure of JUK-8 was analyzed in detail earlier [15,16]. Due to its eight-fold interpenetrated framework, it exhibits breathing behavior upon CO_2_ and H_2_O sorption, accompanied by considerable changes in unit cell volume. Because of these properties, JUK-8 may be regarded as a promising material for construction of gas sensors. A composite film, comprising JUK-8, carbon black and PTFE, was found to change its electrical resistance in line with changes in relative humidity [15]. Recently, an analogous composite film with a Pt catalyst was also used as a logic gate for the simultaneous detection of hydrogen and oxygen gases [17]. 

The aim of this work was to study the co-adsorption of water and selected alcohols (ethanol, 1-propanol, and 2-propanol) in JUK-8. As we plan to explore possible applications of this MOF in the detection of water and/or alcohols, we considered assessment of the possible influence of water vapor on the sorption of alcohols and vice versa to be primarily important [18,19]. 

The co-adsorption study was performed with the use of quasi-equilibrated temperature-programmed desorption and adsorption (QE-TPDA). The QE-TPDA of n-alkanes was introduced and developed by W. Makowski [20] as a method for characterizing micro- and mesoporous materials, including zeolites [21], ordered mesoporous silicas [22], and MOFs [23,24]. In this work, a new experimental QE-TPDA setup, equipped with a non-dispersive infrared detector (NDIR) sensitive to volatile organic compounds, was used for the first time, allowing selective monitoring of temperature-programmed desorption and adsorption of alcohols in the presence of water vapor. 

## 2. Results and Discussion

**Figure 1:**
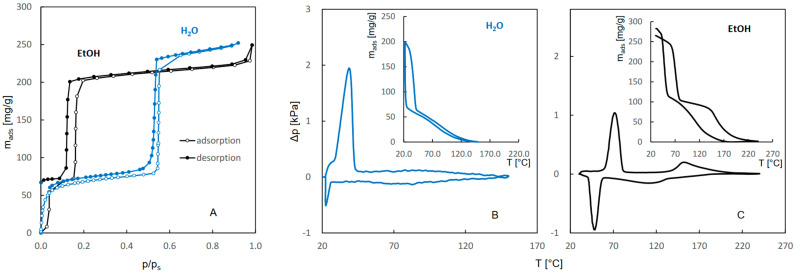
Single component adsorption-desorption isotherms of water and ethanol on JUK-8 (**A**) recorded at 25 °C, compared with the QE-TPDA profiles and the corresponding integral curves obtained for water with TCD detector at heating/cooling rate of 2 °C/min (**B**) and for ethanol with NDIR detector at heating/cooling rate of 2 °C/min (**C**). Sample masses: 3.3 mg (**B**), 5.7 mg (**C**).

**Figure 2:**
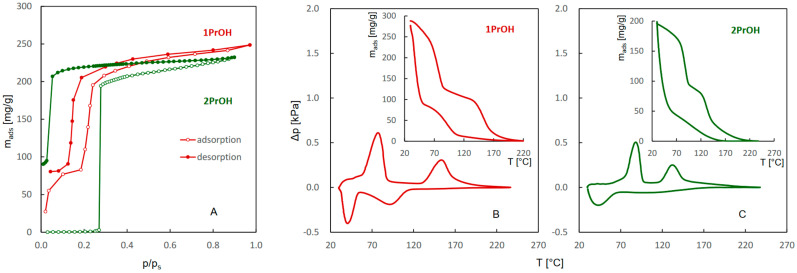
Single component adsorption-desorption isotherms of 1-propanol and 2-propanol on JUK-8 (**A**) recorded at 25 °C, compared with the QE-TPDA profiles and the corresponding integral curves obtained for 1-propanol (**B**) and 2-propanol (**C**) with NDIR detector at heating/cooling rate of 10 °C/min. Sample masses: 7.0 mg (**B**), 7.2 mg (**C**).

The adsorption–desorption isotherms and QE-TPDA profiles of water, ethanol, 1-propanol, and 2-propanol are compared in Figure 1 and Figure 2. The isotherms (Figure 1A and Figure 2A) indicate that sorption of ethanol and 1-propanol follows a two-step pattern, similar to that of water. Although the intensities of the first and second adsorption steps for all of these adsorbates are similar and the locations of the first step overlap, the second adsorption steps observed for ethanol and 1-propanol are shifted to much lower relative pressures. This may indicate that the interactions of alkyl groups with the JUK-8 framework facilitate the transition responsible for breathing behavior. The QE-TPDA profiles of water (Figure 1B) and these alcohols (Figure 1C and Figure 2B,C) also show two-step patterns, consistent with the isotherms. The integral curves, calculated by integration of the desorption and adsorption branches of the QE-TPDA profiles shown in the insets, may be directly compared with the isotherms. They confirm a greater extent of the second adsorption steps and a greater overall affinity of ethanol and 1-propanol for JUK-8, as indicated by higher desorption/re-adsorption temperatures.

The QE-TPDA profiles reveal a remarkable difference between the mechanisms of the high-temperature desorption–adsorption steps of water and alcohols, which are not visible in the isotherms. The broad desorption maximum of water corresponds to the adsorption minimum and are similar in shape. As a consequence, there is no hysteresis in the integral desorption–adsorption profiles of water in the temperature range 40–150 °C. This type of QE-TPDA profile is characteristic of the pore filling mechanism, often observed for rigid frameworks, e.g., zeolites [21]. However, narrow desorption maxima and adsorption minima of ethanol and 1-propanol show considerable hysteresis, which is indicative of sorption-induced phase transition [23,24]. 

The adsorption–desorption isotherms of 2-propanol are considerably different from those observed for ethanol and 1-propanol. Negligible sorption observed in the low-temperature range in the adsorption branch and large adsorption–desorption hysteresis seem to be related to a gate-opening effect [25] that may accompany the structural transformation of the JUK-8 framework. On the other hand, the QE-TPDA profiles of 2-propanol and the corresponding integral curves are more similar to those obtained for 1-propanol. However, even these profiles indicate that adsorption of 2-propanol is delayed compared to 1-propanol.

**Figure 3:**
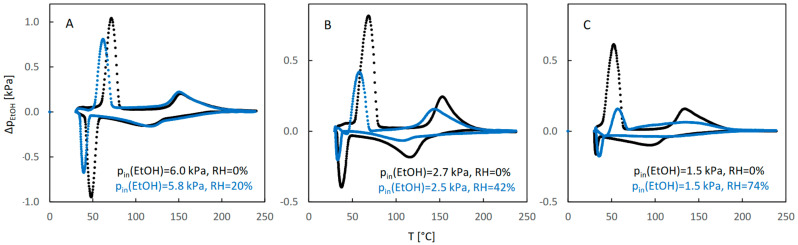
The QE-TPDA profiles of ethanol in JUK-8 in dry Ar (black points) compared with the profiles recorded in presence of water vapor (blue points) at low (**A**), medium (**B**), and high (**C**) relative humidity. Inlet partial pressures of alcohol correspond to high, medium, and low values of the relative pressure (p/p_s_), respectively: A: 66–67%, B: 35–37%, C: 20%. All profiles were recorded using NDIR detector, at heating/cooling rate of 10 °C/min. Sample masses: 5.7 mg (**B**), 7.0 mg (**B**), 5.7 mg (**C**).

The influence of water vapor on the QE-TPDA profiles of ethanol for JUK-8 is presented in Figure 3. At low relative humidity of the carrier gas (20%), the QE-TPDA profile of ethanol is only slightly changed (Figure 3A), with the low temperature maximum and minimum slightly decreased and shifted to even lower temperatures. This indicates that this level of water vapor destabilizes the low-temperature adsorption state, without affecting the high-temperature one. At moderate humidity (ca. 40%) a larger decrease of intensity and shift to lower temperatures are visible for both adsorption states, indicating destabilization extending to the high temperature adsorption state. A further increase of relative humidity (to ca. 70%) leads to a considerable distortion of the QE-TPDA profile, with major changes in the shape and intensity of the peaks.

**Figure 4:**
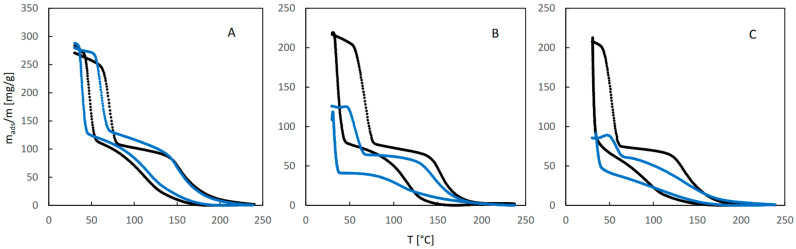
The integral curves calculated from the corresponding QE-TPDA profiles of ethanol in JUK-8 shown in Figure 3.

The suppression of ethanol sorption resulting from the presence of water vapor is better visualized by the integral QE-TPDA profiles shown in Figure 4. While at low relative humidity the sorption capacity of JUK-8 for ethanol is practically unchanged (Figure 4A), the moderate (Figure 4B) and high values (Figure 4C) of RH lead to a substantial decrease of the adsorbed amount (by around 40 and 60%, respectively).

**Figure 5:**
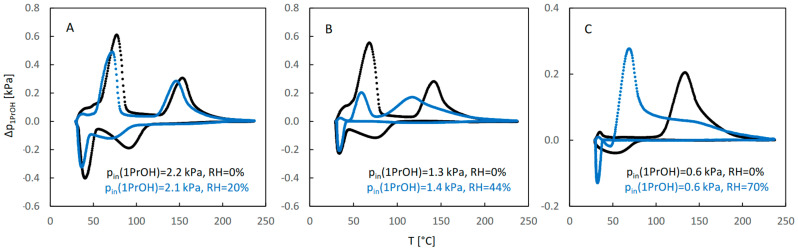
The QE-TPDA profiles of 1-propanol in dry Ar (black points) compared with the profiles recorded in presence of water vapor (blue points) at low (**A**), medium (**B**), and high (**C**) relative humidity. Inlet partial pressures of alcohol correspond to high, medium, and low values of the relative pressure (p/p_s_), respectively: A: 66–69%, B: 41–44%, C: 18%. All profiles were recorded using NDIR detector, at heating/cooling rate of 10 °C/min. Sample masses: 7.0 mg (**A**), 7.0 mg (**B**), 7.0 mg (**C**).

Figure 5 illustrates the influence of water vapor on sorption of 1-propanol. The QE-TPDA profiles of this alcohol observed for a dry carrier gas at high and medium relative pressures (Figure 5A,B) are similar to those of ethanol. Additionally, the effects of water vapor at low and moderate relative humidity are similar to those observed in the case of ethanol. However, both profiles of 1-propanol observed at its low relative pressure (Figure 5A), either for a dry carrier gas or at high relative humidity, are different from those found for ethanol. They have one desorption maximum and one adsorption minimum, the latter shifted to the low temperature range. Apparently, because of the low inlet partial pressure of 1-propanol, the adsorption sites corresponding to the low temperature adsorption state were not occupied. The desorption peak with maximum at 70 °C, observed in the presence of water, should be attributed to the destabilized high-temperature adsorption state. 

**Figure 6:**
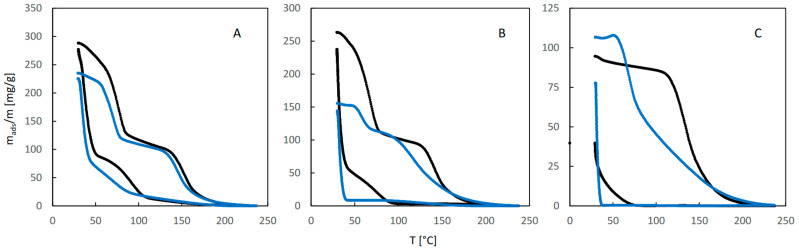
The integral curves calculated from the corresponding QE-TPDA profiles of 1-propanol in JUK-8 shown in Figure 5.

The integral QE-TPDA profiles (Figure 6) reveal considerable desorption–adsorption hysteresis, much larger than in the case of ethanol. It is particularly large at moderate and high relative humidity (Figure 5B,C), where re-adsorption occurs mainly after cooling the sample to the initial temperature (27 °C). 

**Figure 7:**
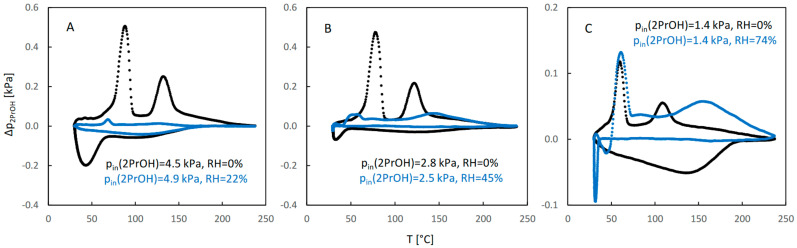
The QE-TPDA profiles of 2-propanol in dry Ar (black points) compared with the profiles recorded in presence of water vapor (blue points) at low (**A**), medium (**B**), and high (**C**) relative humidity. Inlet partial pressures of alcohol correspond to high, medium, and low values of the relative pressure (p/p_s_), respectively: A: 67–73%, B: 37–42%, C: 21%. All profiles were recorded using NDIR detector, at heating/cooling rate of 10 °C/min. Sample masses: 7.2 mg (**A**), 7.2 mg (**B**), 7.2 mg (**C**).

The QE-TPDA profiles of 2-propanol are shown in Figure 7. While the desorption parts of the profiles recorded at high and medium inlet partial pressures of 2-propanol in dry carrier gas (Figure 7A,B) are similar to those observed for ethanol and 1-propanol, the adsorption parts are considerably different—the corresponding minima are broad and shallow and are shifted to lower temperatures. This may indicate that re-adsorption is suppressed, probably due to the gate opening effect experienced by branched molecules of 2-propanol. Even greater differences may be observed for the profiles measured in the presence of water vapor (compared to the corresponding profiles found for ethanol and 1-propanol). These profiles are much less intensive and lack a clear two-step character. The profiles in Figure 7C corresponding to low inlet partial pressure of the alcohol are considerably different from the corresponding profiles of 1-propanol (Figure 5C), as they have retained a distinct two-step character.

**Figure 8:**
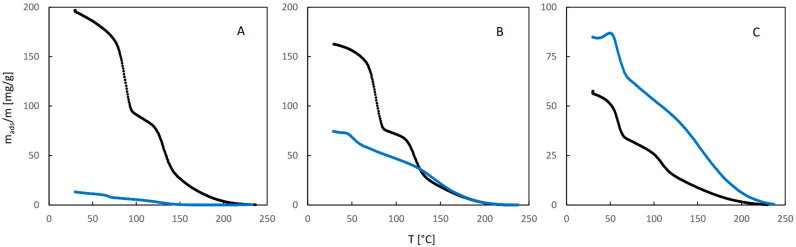
The integral curves calculated from the desorption parts of the corresponding QE-TPDA profiles of 2-propanol in JUK-8 shown in Figure 7.

Due to problems in establishing the baseline of the NDIR signal, resulting from the low intensity of the QE-TPDA profiles of 2-propanol, the corresponding integral curves were considerably distorted, making quantitative analysis of the results difficult. For this reason, only the integral curves calculated from the desorption parts of the profiles are shown in Figure 8. While the shape of the profiles and the trend of their changes with decreasing inlet pressure for desorption of 2-propanol in dry carrier gas are similar to those observed for 1-propanol, the influence of water vapor is remarkably different. It is surprising that for a low RH and high inlet pressure of alcohol, 2-propanol sorption is considerably suppressed. This suppression effect is smaller for medium RH and moderate partial pressure of 2-propanol, while the trend is reversed for high RH and low pressure of the alcohol, i.e., the adsorbed amount of 2-propanol, although small, is larger in the presence of water vapor.

**Figure 9:**
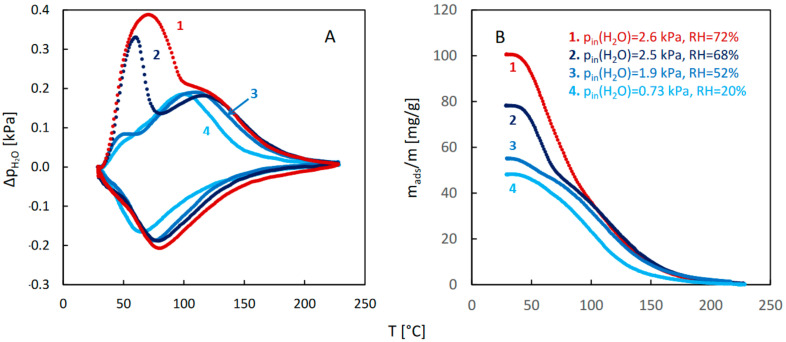
QE-TPDA profiles of H_2_O on JUK-8 observed using RH sensor (**A**), at heating/cooling rate of 10 °C/min and the corresponding desorption integral curves (**B**). Sample mass: 4.4 mg.

The QE-TPDA profiles of water, recorded with the use of a RH sensor (Figure 9), are consistent with the profiles measured using the standard QE-TPDA setup equipped with a TCD detector (Figure 1B). The fact that in the RH-sensor based profiles (Figure 9) the low temperature desorption peaks are not present or are much smaller than in the profiles recorded with the TCD detector, results from the lower inlet pressures of water vapor in the carrier gas. Indeed, recording a complete QE-TPDA profile of H_2_O at saturation conditions (Figure 1B) required using a very low heating/cooling rate and preventing condensation of the water vapor inside the detector. This was not possible in the new experimental system, as indicated by the profile measured for p_in_(H_2_O) = 2.6 kPa (Figure 9A, line 1), which contains a low temperature peak broadened due to condensation. 

Thermodesorption profiles of water from co-adsorption experiments could not be analyzed in the same way as those presented above for the alcohols, because the Ritter MultiGas detector used lacked a selective water vapor sensor. The incorporated RH sensor was also responsive to alcohols, so their presence resulted in convoluted QE-TPDA profiles, combining contributions corresponding to both adsorbates. Despite this, the RH signal profiles may be analyzed qualitatively using the results obtained for pure water vapor (Figure 9) as a reference. Such analysis may provide some insights into thermodesorption of water, which occurs along with thermodesorption of an alcohol. 

**Figure 10:**
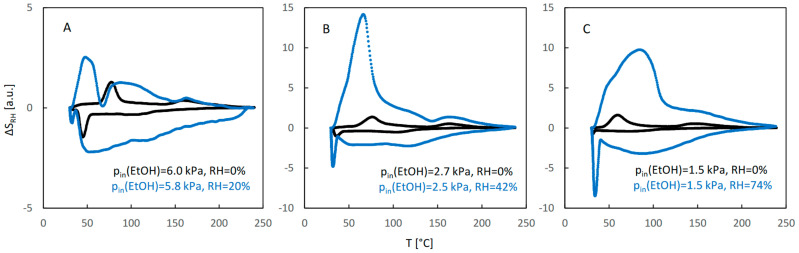
Evolution of RH signal observed during thermodesorption of ethanol (black points) and ethanol and water (blue points) at low (**A**), medium (**B**), and high (**C**) relative humidity, corresponding to the QE-TPDA profiles from Figure 3.

**Figure 11:**
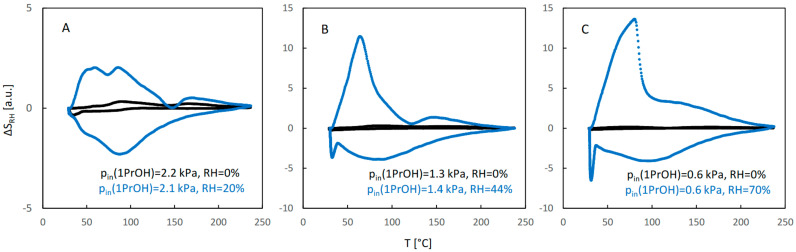
Evolution of RH signal observed during thermodesorption of 1-propanol (black points) and 1-propanol and water (blue points) at low (**A**), medium (**B**), and high (**C**) relative humidity, corresponding to the QE-TPDA profiles from Figure 5.

**Figure 12:**
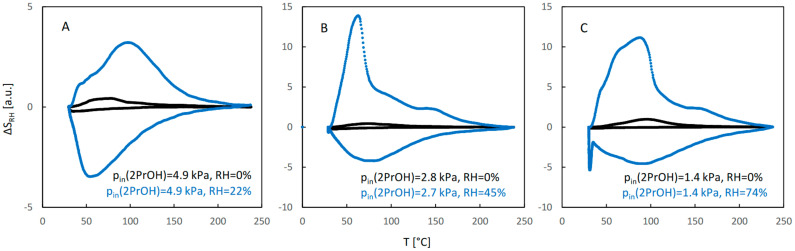
Evolution of RH signal observed during thermodesorption of 2-propanol (black points) and 2-propanol and water (blue points) at low (**A**), medium (**B**), and high (**C**) relative humidity, corresponding to the QE-TPDA profiles from Figure 7.

The profiles of the RH signal observed during QE-TPDA of pure alcohols or water–alcohol mixtures are shown in Figure 10, Figure 11 and Figure 12. They correspond exactly to the profiles of alcohols shown in Figure 3, Figure 5 and Figure 7. It may be noticed that only for high relative partial pressures of ethanol and 1-propanol (Figure 10A and Figure 11A) is the intensity of the RH profiles of pure alcohols comparable to that of the RH profiles observed for water–alcohol mixtures. Actually, interpretation of these profiles is more difficult. It is not clear whether the visible distortions of the RH signal result only from competitive adsorption and desorption of both adsorbates, without being affected by their individual and mutual interactions with the RH sensor. On the other hand, the profile recorded under these conditions for 2-propanol (Figure 12A) is similar to that of pure water. This confirms the findings resulting from the NDIR QE-TPDA profiles of 2-propanol, indicating that at 20% RH its sorption is considerably suppressed. 

All RH profiles recorded at medium relative pressures of both water and alcohol (Figure 10B, Figure 11B and Figure 12B) contain sharp maxima at ca. 70°C. The lack of such a maximum in the corresponding QE-TPDA profile of pure water (Figure 9A, curve 3) indicates that the sorption of water is enhanced by the presence of alcohols. Most probably, after opening of the JUK-8 framework, the available space is filled with a quasi-liquid mixture of both adsorbates. In the case of high levels of RH and low relative pressures of alcohols, the RH profiles are similar to those recorded for pure water at 72% RH. This indicates that the presence of alcohols does not considerably affect the thermodesorption of water, apart from the re-adsorption step, which occurs at slightly higher temperatures. This again confirms that the presence of alcohols stabilizes the low-temperature adsorption of water.

## 3. Materials and Methods

JUK-8 and 4-pyridyl functionalized benzene-1,3-dicarbohydrazide (pip) were synthesized according to the method published earlier [15]. The pip linker was obtained in a condensation reaction carried out under reflux in ethanol between isophthalic acid dihydrazide and pyridine-4-carboxaldehyde (1:2 ratio). JUK-8 was synthesized by heating stoichiometric amounts (1:1:1) of 4,4′-oxybis(benzenedicarboxylic) acid (H_2_oba), pip, and Zn(NO_3_)_2_·6H_2_O dissolved in DMF/H_2_O (9:1 *v*/*v*) at 80 °C for 24 h. Demineralized water as well as analytical grade reagents and solvents (Sigma-Aldrich, St. Louis, MO, USA, POCh/Avantor, Polmos) were used in the synthesis and/or sorption measurements without further purification. 

Static measurements of the adsorption–desorption isotherms of water and ethanol at 25 °C were performed using the Autosorb iQ MP apparatus (Quantachrome/Anton Paar, Boynton Beach, FL, USA).

The QE-TPDA profiles were recorded in a new experimental system, comprising two mass flow controllers (Brooks Instrument, Hatfield, PA, USA), two saturators, a mixing chamber, a sample tube placed inside a coiled heating element serving as a furnace, and a MultiGas detector (Ritter) equipped with a non-disperse infrared module, sensitive to volatile organic compounds, and with a humidity sensor. Argon (purity 5.0) was used as the carrier gas at a flow rate of 16.6 cm^3^/min. The QE-TPDA experiments were performed by cyclic heating and cooling of the sample placed in the stream of the carrier gas containing the admixture of alcohol and/or water vapor. 

For comparison purposes, the standard QE-TPDA setup [20,21,22,23], equipped with a thermal conductivity detector (TCD), was also used in water thermodesorption measurements. In this system, helium was used as a carrier gas at a flow rate of 8 cm^3^/min. 

## 4. Conclusions

In this study, a new experimental setup for quasi-equilibrated temperature programmed desorption and adsorption (QE-TPDA) measurements, equipped with an NDIR detector sensitive to volatile organic compounds, was presented for the first time. The measurements performed using this setup were successfully applied to study the influence of water vapor on the sorption of C_2_-C_3_ alcohols in a selected flexible MOF—JUK-8. The QE-TPDA profiles of alcohols confirmed two-step adsorption–desorption patterns, which were also visible in the corresponding isotherms. 

The results of this study show a complex influence of water on the sorption of the alcohols studied. For ethanol and 1-propanol, low levels of relative humidity resulted in a slight destabilization of the low-temperature adsorption state, while for 2-propanol, almost complete suppression of adsorption was observed. For medium levels of RH, the thermodesorption profiles of ethanol and 1-propanol indicated a further decrease in adsorption, but an increase in the case of 2-propanol. It was found that moderate relative pressures of alcohols enhanced the low-temperature sorption of water. 

The outcome of this work indicates that the possible application of JUK-8 in sensing technology is problematic since this MOF is not a selective sorbent of water or alcohols. On the other hand, the results presented here show the potential of the QE-TPDA approach in studies of co-adsorption of water and volatile organic compounds. However, further work aimed at developing and testing selective and sensitive water vapor sensors seems necessary.

## Data Availability

Experimental adsorption–desorption isotherms and profiles of quasi-equilibrated temperature-programmed desorption and adsorption of water, ethanol, 1-propanol, and 2-propanol on JUK-8 are openly available in the Jagiellonian University Repository (https://doi.org/10.57903/UJ/YKJAYR, accessed on 1 April 2024).
